# HMSCs exosome‐derived miR‐199a‐5p attenuates sulfur mustard‐associated oxidative stress via the CAV1/NRF2 signalling pathway

**DOI:** 10.1111/jcmm.17803

**Published:** 2023-06-29

**Authors:** Chuchu Gong, Zhengyan Gu, Xinkang Zhang, Qingqiang Xu, Guanchao Mao, Zhipeng Pei, Wenqi Meng, Jinfeng Cen, Jihao Liu, Xiaowen He, Mingxue Sun, Kai Xiao

**Affiliations:** ^1^ Lab of Toxicology and Pharmacology, Faculty of Naval Medicine Naval Medical University Shanghai People's Republic of China; ^2^ Medical Care Center, Naval Medical Center of PLA Naval Medical University Shanghai China; ^3^ Basic Medical Center for Pulmonary Disease Naval Medical University Shanghai People's Republic of China; ^4^ Department of Pharmaceutical Sciences, School of Pharmacy Naval Medical University Shanghai People's Republic of China; ^5^ Origincell Technology Group Co., Ltd. Shanghai People's Republic of China

**Keywords:** acute lung injury, exosomes, human umbilical cord mesenchymal stem cells, miRNA‐199a‐5p, oxidative stress, sulfur mustard

## Abstract

Sulfur mustard (SM) is a blister‐producing chemical warfare agent which could lead to a cascade of systemic damage, especially severe acute lung injury. Oxidative stress is considered to be vital processes for the SM toxicity mechanism. We previously proved the therapeutic effect of exosomes derived from bone marrow mesenchymal stromal cells in promoting the repair of alveolar epithelial barrier and inhibiting apoptosis. However, the key functional components in exosomes and the underlying mechanisms have not been fully elaborated. This research shed light on the function of the key components of human umbilical cord mesenchymal stem cell‐derived exosomes (HMSCs‐Ex). We noted that HMSCs‐Ex‐derived miR‐199a‐5p played a vital role in reducing pneumonocyte oxidative stress and apoptosis by reducing reactive oxygen species, lipid peroxidation products and increasing the activities of antioxidant enzymes in BEAS‐2B cells and mouse models after exposure to SM for 24 h. Furthermore, we demonstrated that the overexpression of miR‐199a‐5p in HMSCs‐Ex treatment induced a further decrease of Caveolin1 and the activation of the mRNA and protein level of NRF2, HO1 and NQO1, compared with HMSCs‐Ex administration. In summary, miR‐199a‐5p was one of the key molecules in HMSCs‐Ex that attenuated SM‐associated oxidative stress via regulating CAV1/NRF2 signalling pathway.

## 1 INTRODUCTION

Sulfur mustard (2,2‐dichlorodiethyl sulfide, SM), a vesicant causing heavy casualties,^1^, ^2^ is still a threat as an agent of warfare/terrorism nowadays. Exposure to SM can lead to severe injury of multiple organs and systems including respiratory system, skin and eyes.^3^, ^4^ The leading cause of mortality and morbidity among them is considered to be severe acute lung injury (ALI).^5^ The exact molecular mechanism of the toxic action of SM remains unclear. However, oxidative stress and inflammatory reaction are regarded as the most relevant pathological consequences.^6^ Despite decades of intense research, current therapy for SM exposure is far from optimal.^7^

Recently, exosomes have attracted massive interest for their mediatory role in communication between cells.^8^, ^9^ Exosomes are lipid bilayer membranous vesicles with a diameter ranging from about 40–160 nm and an average of about 100 nm.^10^ They are secreted by a variety of cells. They can fuse with the cell membrane of neighbouring cells through exocytosis to carry out information transmission, regulate inter‐cell signal transmission, and perform a variety of biological functions including anti‐inflammatory and antioxidant activities.^11^, ^12^, ^13^ At present, it is generally believed that the functional significance of exosomes depend on the contents of exosomes.^9^ These components include proteins, lipids, cytokines or genetic materials. In particular, studies have shown that microRNAs (miRNAs), delivered by exosomes, play significant roles in various pathological and physiological processes, such as immune regulation, epigenetic modification, body development, tumour progression and so forth.^14^, ^15^ Since the main components in exosomes are miRNAs, exosomes play important regulatory roles in treating various disease by delivering exosomal miRNAs.^16^

Previously, our studies showed that bone marrow‐derived mesenchymal stem cells have anti‐inflammatory, immunomodulatory and pro‐reparative effects on lung injury in mice due to SM exposure.^17^ Also, bone marrow‐derived mesenchymal stromal cell‐derived exosomes (BMSCs‐Ex) have a significant role in coordinating the repair of SM‐induced lung epithelial barrier injuries.^18^ However, the key functional components of exosomes and the mechanisms are not sufficiently elaborated. In recent years, human umbilical cord mesenchymal stem cells (HMSCs) are considered as an ideal cell‐based therapy for various diseases because of the advantages such as abundant sources, no influence on the donor, easy collection and transportation.^19^ Thus, we hypothesized that the administration of HMSCs‐derived exosomes (HMSCs‐Ex) might provide a resultful treatment for ALI induced by SM and become a prospective tool for clinical practice.

In this study, we explored the effect of HMSCs‐Ex on SM‐induced lung injury. First, the influence of HMSCs‐Ex on the survival rate and pulmonary function were assessed. Then, we evaluated whether HMSCs‐Ex alleviated SM‐induced lung injuries by suppressing oxidative stress and apoptosis. Further, we examined whether HMSCs‐Ex exerted anti‐oxidative stress and anti‐apoptotic effects through the delivery of miR‐199a‐5p in vitro and in vivo, and finally, we verified these effects via inhibiting the nuclear factor erythroid 2‐related factor 2 (NRF2) signalling pathway. This study provides solid evidence and a deeper understanding of the functional significance of HMSCs‐Ex in treating ALI.

## 2 MATERIALS AND METHODS

### 2.1 Cells and mice

BEAS‐2B cells (ATCC, CRL‐9609) were cultured with DMEM (Wisent) containing 10% foetal bovine serum (FBS) (Hyclone) and 1% penicillin–streptomycin (Gibco) at 37°C in 5% CO_2_. Human lung fibroblasts (HFLs; ATCC, CRL‐153) were cultured in minimal essential medium alpha (α‐MEM; Gibco‐BRL, Life Technologies) supplemented with 10% exosomes‐deprived FBS (Invitrogen) obtained by centrifugation at 100,000× ***g*** at 37°C in 5% CO_2_. BEAS‐2B cells and HFLs cells were purchased from the Chinese Academy of Sciences and tested for mycoplasma contamination. HMSCs were donated by Origincell Technology Group Company and the HMSCs‐conditioned medium consisted of mesenchymal stem cell basal medium (Dakewe) supplemented with 5% animal serum‐free, xenogeneic‐free cell culture supplements (EliteCell, EPG‐500).

### 2.2 Isolation of exosomes and miR‐199a‐5p pretreatment of HMSCs‐Ex

The exosomes were isolated and purified by differential ultracentrifugation from the supernatant of HMSCs and HFLs. Briefly, when the HMSCs and HFLs were cultured at 3–8 passages and reached 80%–90% confluence, they were cultured in the conditioned medium with 5% CO_2_ at 37°C for 48 h. Subsequently, the supernatants containing exosomes were harvested. The supernatants were centrifuged to remove cell debris at 2000 ***g*** for 10 min at 4°C and subsequently passed through a 0.22 μm filter. The cleared supernatant was centrifuged at 120,000 ***g*** for 70 min three times after transferred to a new glass tube. The exosomes underwent another centrifuge at 110,000 ***g*** for 70 min and resuspended in PBS and then passed through 0.22 μm sterile filters. All steps were performed at 4°C. Finally, the Piercebicinchoninic acid (BCA) assay kit (Thermo Fisher Scientific) was used to determine the concentration of exosomes. HSP70 (Abcam, ab53496), CD63 (Abcam, ab217345) and TSG101 (Santa Cruz, sc‐7964) were detected by western blotting. Mimics of miR‐199a‐5p and the negative control (miR‐NC) were synthesized by Ribo Biotech and transfected into HMSCs‐Ex using the Exo‐Fect exosome reagent (System Biosciences). The exosomes were stored at minus 80°C.

### 2.3 Transmission electron microscopy analysis

Briefly, 10 μL of the freshly isolated exosomes was dropped onto a copper grid covered with Formvar in chloroform at room temperature for 5 min. The excess liquid was absorbed with a clean absorbent paper. The grids were washed with sterile distilled water and subsequently stained with 1% uranyl acetate in ddH_2_O for 3–5 min. After removing the excess liquid and drying the mesh for 2 min under an incandescent lamp, the copper mesh was analysed using a Hitachi H‐7650 transmission electron microscope (Hitachi Company) at 80 kV.

### 2.4 Construction of CAV1 overexpressed BEAS‐2B stable cell lines

To construct overexpressing CAV1 cell lines, full‐length CAV1 (NM_001172895) was cloned into a lentiviral packaging system (Obio Technology) and transfected into BEAS‐2B cell lines according to the manufacturer's instructions. To select the stable transduced cells, puromycin at the concentration of 10 μg/mL was used. GFP were used for confirmation. Cells were confirmed by fluorescence microscopy and western blotting.

### 2.5 ALI mouse model and administration of HMSCs‐Ex

As previously described, the concentration of SM was 40 mg/kg when used in determining the survival rate and respiratory function experiments and 30 mg/kg when conducting other experiments. Before use, SM was formulated with a propanediol solution (Sigma) and the volume of the injection was 0.05 mL/10 g in all mice. The final concentration of exosomes applied for treating lung injury was 20 mg/mL for each animal. NAC was used as a positive control in our study since it is accepted as a potential antioxidant against SM‐induced toxicity.^20^ NAC was dissolved to the final concentration of 100 μM in PBS in the in vitro experiment. The mice were randomly assigned into five groups (*n* = 8 each) to investigate the effects of HMSCs‐Ex in improving the survival rate and respiratory function in SM‐exposed models: (i) the CTRL group, subcutaneous injection of propanediol solution (same volume as SM); (ii) the SM group, subcutaneous injection of SM; tail vein injection of PBS 30 min after the injection of SM; PBS were given on the first day and the third day two times; (iii) the HMSCs‐Ex group, tail vein injection of HMSCs‐Ex (3 × 10^8^ particles, resuspended in 150 μL of PBS) 30 min after the injection of SM; HMSCs‐Ex were given two times, that is on the first day and the third day, respectively; (iv) the NAC group, intraperitoneal injection of NAC (200 mg/kg) 30 min after the injection of SM; NAC was given once a day (five times altogether); (v) and the HFLs‐Ex group, tail vein injection of HFLs‐Ex (3 × 10^8^ particles, resuspended in 150 μL of PBS) 30 min after the injection of SM. HFLs‐Ex were injected once a day on the first day and the third day. The mice were randomly distributed into the following groups (*n* = 8 each) to determine whether HMSC‐Ex‐derived miR‐199a‐5p was involved in SM‐induced oxidative stress reaction and apoptosis by targeting CAV1/NRF2 pathway: the CTRL group, the SM group, the HMSCs‐Ex group, the miR‐199a‐HMSCs‐Ex group (3 × 10^8^ particles, resuspended in 150 μL of PBS) and the miR‐NC‐HMSCs‐Ex group (3 × 10^8^ particles, resuspended in 150 μL of PBS). After SM exposure, the mice were either excised for evaluation or sacrificed by injecting 4 mL/kg chloral hydrate. After opening the chest, the lung tissue samples were collected for analysis. Saline was used to rinse the lungs and then removed immediately. Part of the samples was fixed in 4% buffered formaldehyde for histopathologic evaluation and immunohistochemistry. The other part was snap‐frozen in liquid nitrogen and stored at minus 80°C until analysis.

### 2.6 Measurement of antioxidant enzyme activity and lipid peroxidation

Lung tissues or cells samples were collected after multiple treatments, sonicated in cold PBS and centrifuged at 10,000 rpm at 4°C for 10 min. The supernatant was used for MDA and antioxidant enzyme assays detected with commercial kits (Jiancheng) following the manufacturer's protocols. BCA kit was used to determine the protein content.

### 2.7 Measurement of intracellular reactive oxidative species

SM‐induced ROS in mice and BEAS‐2B cells were detected by dihydroethidium (DHE) and DCFH‐DA staining, respectively. DHE staining in vivo was performed following the manufacturer's protocols (FUJIFILM Wako Pure Chemical Corporation). After the indicated administration, fresh lung tissues were harvested, and 5‐μm sections were cut in each group. Diluted with dimethyl sulfoxide (DMSO), the DHE stain solution was added to each tissue section and incubated for 30 min at 37°C in a humidified chamber. Then, a fluorescence microscope (BioTek) was used to detect red fluorescence signal. DCFH‐DA staining in vitro was conducted using a DCFH‐DA probe following the manufacturer's protocols (Beyotime). After multiple treatments, the BEAS‐2B cells were washed with PBS gently and then incubated with 10 μM DCFH‐DA in the dark for 20 min at room temperature. Then, the BEAS‐2B cells were washed with PBS again, and a fluorescent microscope (BioTek) was applied to determine the DCF fluorescence images. The ROS level was presented as fluorescence intensity per unit area.

### 2.8 Evaluation of the survival rate

Healthy adult male ICR mice (20–25 g) were purchased from the Experimental Animal Centre of Naval Medical University. Before the experiment, the mice were acclimated for 7 days. The animal were used and cared in accordance with protocols approved by the Institutional Animal Care and Use Committee of Naval Medical University. The mice were divided into five different groups randomly. The general conditions including body weight, food and water intake and condition of urination and defecation were recorded and carefully compared for 14 days. The primary endpoint of the study was the survival of the mice: treated versus untreated control.

### 2.9 Detection of BALF protein and wet‐to‐dry lung weight ratio

The mice were inserted with a small‐calibre cannula into the trachea and fixed by a 2/0 suture after anaesthetised with peritoneal pentobarbitone. The lungs were washed with 800 μL of PBS for three times, and the PBS was collected to sterile Eppendorf tubes at 37°C for more than 1 h. Then, the tubes were processed to centrifuge at 800 ***g*** for 15 min. The supernatant was collected and transferred to new sterile Eppendorf tubes at minus 80°C. The total protein in the supernatant was measured using a BCA protein assay kit. The optical density (OD) was detected at 562 nm using amicroplate reader (BioTek).

For the analysis of W/D ratio, the mice chests were cut following sacrifice and lung tissues were excised. After removing blood from the surface, the wet lung tissues were weighed. The dry weight of the lung tissues was obtained after being dried in an electrothermal oven at 72°C for 72 h. The W/D ratio was calculated and used as an index to detect the lung oedema.

### 2.10 Histopathological staining

After fixation in 10% paraformaldehyde for 24 h, the lung tissues were dehydrated in serial concentrations of ethanol, dealt with the xylene clearing agent, and embedded in paraffin. The tissues were cut into sections in 5 μm and then subjected to H&E staining, followed by microscopic imaging under ×400 magnifications.

The histological examinations were carefully assessed by a qualified pathologist. According to a scoring criteria published previously, scores ranging from 0 (normal) to 4 (severe) were assigned to the degree/abundance of alveolar wall thickening, alveolar oedema, inflammation and haemorrhage of alveoli and interstitium, necrosis, atelectasis, and pseudo membrane formation.^21^ The sum of all the above‐mentioned scores of each mouse was calculated as the total injury score to reveal the extent of injury.

### 2.11 Immunohistochemistry staining

The molecular changes in CAV1 and NRF2 levels and oxidative stress index (8‐OHdG) levels were measured by immunohistochemistry. After deparaffinization, the tissue sections were soaked in a 3% H_2_O_2_ solution for 15 min. Subsequently, the sections were washed in PBS and antigen retrieved using 0.1 M sodium citrate. After incubation with 3% bovine serum albumin (BSA) (Sigma‐Aldrich) at 37°C for 30 min, the tissues were added and incubated with antibody to CAV1 (sc‐53564, Santa Cruz), NRF2 (CY1851, Abways), and 8‐OHdG (sc‐66036, Santa Cruz) overnight at 4°C, respectively. Then, the sections were subsequently treated with secondary antibodies at room temperature for 30 min on the next day. The nuclei were stained with 4′,6‐diamidino‐2‐phenylindole (DAPI; Sigma‐Aldrich) and incubated for 10 min. The sections were evaluated with a bright‐field microscope (BioTek). Ten microscopic fields were randomly chosen and counted according to the positive number and staining intensity of each specimen.

### 2.12 Terminal deoxynucleotidyl transferase enzymatic dUTP nick end labelling staining

A TUNEL protocol kit (Roche) was applied to detect genomic DNA breakage during apoptosis in lung tissues. Following deparaffinization and antigen retrieval, permeabilization of the lung tissue was performed with Triton X‐100 (ST795, Beyotime), followed by a subsequent incubation with 30 μL of TUNEL reaction mixture in the dark at room temperature for 1 h. Finally, the samples were stained with DAPI (Sigma‐Aldrich). Further, 10 fields of each group were chosen randomly under a fluorescence microscope (BioTek) and ImageJ software was used to calculate the average fluorescence intensity.

### 2.13 Flow cytometry analysis

For the identification of HMSCs, extracellular surface proteins were detected by flow cytometry (Beckman CytoFlex). The HMSCs were subjected to antibodies against FITC‐CD44 (R&D Systems, FAB3660P), FITC‐CD29 (Invitrogen, 12‐0299‐42), FITC‐CD73 (Invitrogen, 17‐0739‐42), FITC‐CD166 (Invitrogen, 12‐1668‐42), FITC‐CD34 (Invitrogen, MA1‐10202), FITC‐CD45 (Invitrogen, 58‐0459‐42) and FITC‐CD11b (R&D Systems, FAB16991P), respectively, at room temperature for 30 min. Then, the treated cells were centrifugated and washed with PBS twice and suspended in FACS buffer for flow cytometry analysis. For the apoptosis analysis of the BEAS‐2B cells, SM‐treated cells were incubated with miR‐NC‐HMSCs‐Ex or HMSCs‐Ex or miR‐199a‐HMSCs‐Ex for 24 h. Following the instructions of an AV/PI Apoptosis Detection Kit (BD Biosciences), the collected cells were resuspended in binding buffer and incubated with Annexin V–fluorescein isothiocyanate for 15 min and PI at room temperature for 15 min in the dark. FlowJo Software was applied to measure the cell apoptosis data.

### 2.14 Cell viability assay

The CCK‐8 (Dojindo) was applied to measure the cell viability. In brief, the cells were cultured at a density of 5000 cells per well in a 96‐well plate. To evaluate the IC_50_ value of SM on BEAS‐2B cells, the cells were exposed to different concentrations (0, 12.5, 25, 50, 100 and 200 μM) of SM for 30 min. Then, the cells were incubated with new culture medium for 24 h. For the assessment of the effect of HMSCs‐Ex on SM‐exposed cell viability, HMSCs‐Ex with concentrations of 1 × 10^9^ particles resuspended in 150 μL of PBS were added to BEAS‐2B cells after exposure to SM at the concentration of 12.5 μM. After incubation for 24 h, cell viability assay was applied. For the analysis of miRNA inhibitors on cell viability, 10 inhibitors of miRNAs (miR‐100‐5p, miR‐21, miR‐23a‐3p, let‐7a‐5p, miR‐145‐5p, miR‐424‐5p miR‐16‐5p, miR‐24‐3p, miR‐199a‐5p and miR‐15a‐5p) and the control (miR‐NC) were synthesized by Ribo Biotech. The inhibitors were transfected into BEAS‐2B cells using lipofectamine RNAiMAX (Invitrogen) for 48 h. Then, SM was exposed to the cells at the concentration of 12.5 μM and treated with HMSCs‐Ex for 24 h. The CCK‐8 assay was conducted by co‐culturing with 10 μL of CCK‐8 solution and 100 μL of culturing medium at 37°C for 1 h. A microplate reader (BioTek) was applied to quantify the absorbance of the solution by OD values at 450 nm.

### 2.15 Hoechst 33342 and JC‐1 staining

SM‐exposed BEAS‐2B cells were treated with HMSCs‐Ex or NAC or HFLs‐Ex for 24 h in six‐well plates as described earlier. The cells were then incubated with 50 mg/mL Hoechst 33342 (Sigma‐Aldrich) or 5 mg/mL JC‐1 (Beyotime) reaction mixture at room temperature for 20 min following the manufacturer's protocols. For the Hoechst 33342 staining experiment, the typical morphology of the cells was obtained, of which the apoptotic cells possessing the characteristic of nuclear condensation and/or fragmentation, the shrinking of cytoplasm, and membrane blebbing. For the JC‐1 staining assay, the increased green relative to red fluorescent intensity ratio representing the depolarization of the mitochondrial membrane potential was measured. Fluorescence pictures were taken by a fluorescent microscope (BioTek) and analysed using open‐source software plugins for ImageJ software.

### 2.16 Dual‐luciferase reporter assay

The bioinformatics website (TargetScan, https://www.targetscan.org/) was applied to predict the target gene of miR‐199a‐5p and CAV1 was selected as a candidate target. The wild‐ and mutant‐type sequences (mutation at the binding site of miR‐199a‐5p) of CAV1 3′‐untranslational region (3′UTR) were produced and amplified to construct pmir‐GlO‐CAV1 3′UTR wild‐type and pmir‐GlO‐CAV1 3′UTR mutant‐type recombinant reporter vectors, named as CAV1‐WT and CAV1‐MUT, respectively. After miR‐199a‐5p or miR‐NC and the recombinant vector (CAV1‐WT or CAV1‐MUT) were transfected to BEAS‐2B cells for 48 h, the relative luciferase activity was measured applying the dual‐luciferase assay system (Promega).

### 2.17 Immunofluorescence staining and confocal imaging

After the cell fixation and permeabilization procedure, the cells were blocked in 5% BSA and subsequently incubated in the anti‐CAV1 (sc‐53564, Santa Cruz) and anti‐NRF2 primary antibodies (CY1851, Abways) overnight at 4°C. On the next day, the cells were washed three times with PBS and incubated in the dark with fluorescence‐labelled secondary antibodies for 1 h. After 3× PBS wash, nuclei staining with DAPI (Sigma‐Aldrich) was conducted for 5 min. For confocal imaging, the fluorescence images were obtained with a confocal laser scanning microscope (Carl Zeiss) and the fluorescence intensity was processed and analysed using ZEISS ZEN Imaging Software (Carl Zeiss).

### 2.18 Western blot analysis

The treated lung tissues or BEAS‐2B cells were lysed in radio‐immunoprecipitation assay (RIPA) buffer for 20 min, and supernatants were collected. The BCA assay kit was applied to determine the concentrations of proteins. Further, 20 μg proteins were loaded into the lanes of 10% sodium dodecyl sulfate–polyacrylamide gel electrophoresis (SDS‐PAGE) before electroblotting onto polyvinylidene fluoride (PVDF) membranes (Bio‐Rad). After blocked with 3% BSA (Sigma‐Aldrich) at room temperature for 1 h, the membranes were incubated with antibodies against CD9 (Abcam, ab223052), CD61 (R&D, AF2266), CD63 (Abcam, ab217345), CAV1 (Abcam, ab84811), NRF2 (Abcam, ab62352), HO1 (Protein Tech, 27282‐1‐AP), NQO1 (Proteintech, 67240‐1‐Ig), Histone H3 (Abcam, ab176842) and β‐Tubulin (Sigma, SAB4500088) overnight at 4°C. After rinsing, the membranes were then incubated with a HRP‐labelled secondary antibodies that binds primary antibody for 1 h. Finally, the membranes were scanned using electrochemiluminescence (ECL; Millipore) and quantified using ImageJ software.

### 2.19 Reverse transcriptase–polymerase chain reaction

TRIzol (Takara) and DNase (Invitrogen) were applied to isolate total RNA and remove genomic DNA from BEAS‐2B cells and lung samples. Following the manufacturer's instructions of the reverse transcriptase kit (Takara), cDNA was synthesized in TProfessional Thermocycler (Biometra). The primers were synthesized as follows: HO1 (Human), 5′‐CTGACCCATGACACCAAGGAC‐3′ and 5′‐AAAGCCCTACAGCAACTGTCG‐3′; NQO1 (Human), 5′‐GGCAGAAGAGCACTGATCGTA‐3′ and 5′‐TGATGGGATTGAAGTTCATGGC‐3′; NRF2 (Human), 5′‐GCCTGTAAGTCCTGGTCATCGG‐3′ and 5′‐ACTGCTCTTTGGACATCATTTCGTT‐3′; CAV1 (Human) 5′‐TGTTTTGCTCCTGATCTGA‐3′ and 5′‐TCGGGGAATTCAATACTCA‐3′; GAPDH (Human), 5′‐CGGATTTGGTCGTATTGGG‐3′ and 5′‐CTGGAAGATGGTGATGGGATT‐3′; HO1 (Mouse), 5′‐TCCTTGTACCATATCTACACGG‐3′ and 5′‐GAGACGCTTTACATAGTGCTGT‐3′; NQO1 (Mouse), 5′‐AGGATGGGAGGTACTCGAATC‐3′ and 5′‐AGGCGTCCTTCCTTATATGCTA‐3′; NRF2 (Mouse), 5′‐TCTTGGAGTAAGTCGAGAAGTGT‐3′ and 5′‐GTTGAAACTGAGCGAAAAAGGC‐3′; CAV1 (Mouse) 5′‐GGCACTCATCTGGGGCATTTA‐3′ and 5′‐CTCTTGATGCACGGTACAACC‐3′; GAPDH (Mouse), 5′‐AGGCCGGTGCTGAGTATGTC‐3′ and 5′‐TGCCTGCTTCACCACCTTCT‐3′; U6 (Human), 5′‐AAAGCAAATCATCGGACGACC‐3′ and 5′‐GTACAACACATTGTTTCCTCGGA‐3′; U6 (Mouse), 5′‐ATGGCGGACGACGTAGATCA‐3′ and 5′‐AGCTCTCGGTCATTTCTCATTTT‐3′. GAPDH was used as an internal control. qRT‐PCR was used to compare the relative mRNA expression levels with SYBR Green I system using TB Green Premix Ex Taq II (Takara) in ABI 7300 (Applied Biosystems Inc.) machine. To assess miRNA expression, TaqMan miRNA assays (TransScript) were applied to quantify relative miR‐199a‐5p expression levels, using RNU6‐1 (U6) small nuclear RNA as an internal control. A mean CT value of each sample was compared using the comparative cycle threshold (ΔΔCt) method.

### 2.20 Statistical analysis

All data were presented in the form of the mean ± standard deviation. Statistical evaluations was performed using the Student *t*‐test for two groups comparisons or one‐way analysis of variance (ANOVA) for multiple groups comparisons. SPSS software Version 21.0 (SPSS Inc.) was applied for statistical analysis. Values of *p* < 0.01 and *p* < 0.001, indicated by two (**) and three (***) asterisks, were considered statistically significant differences.

## 3 RESULTS

### 3.1 HMSCs‐Ex rescued SM‐induced lung injury in vivo

**FIGURE 1:**
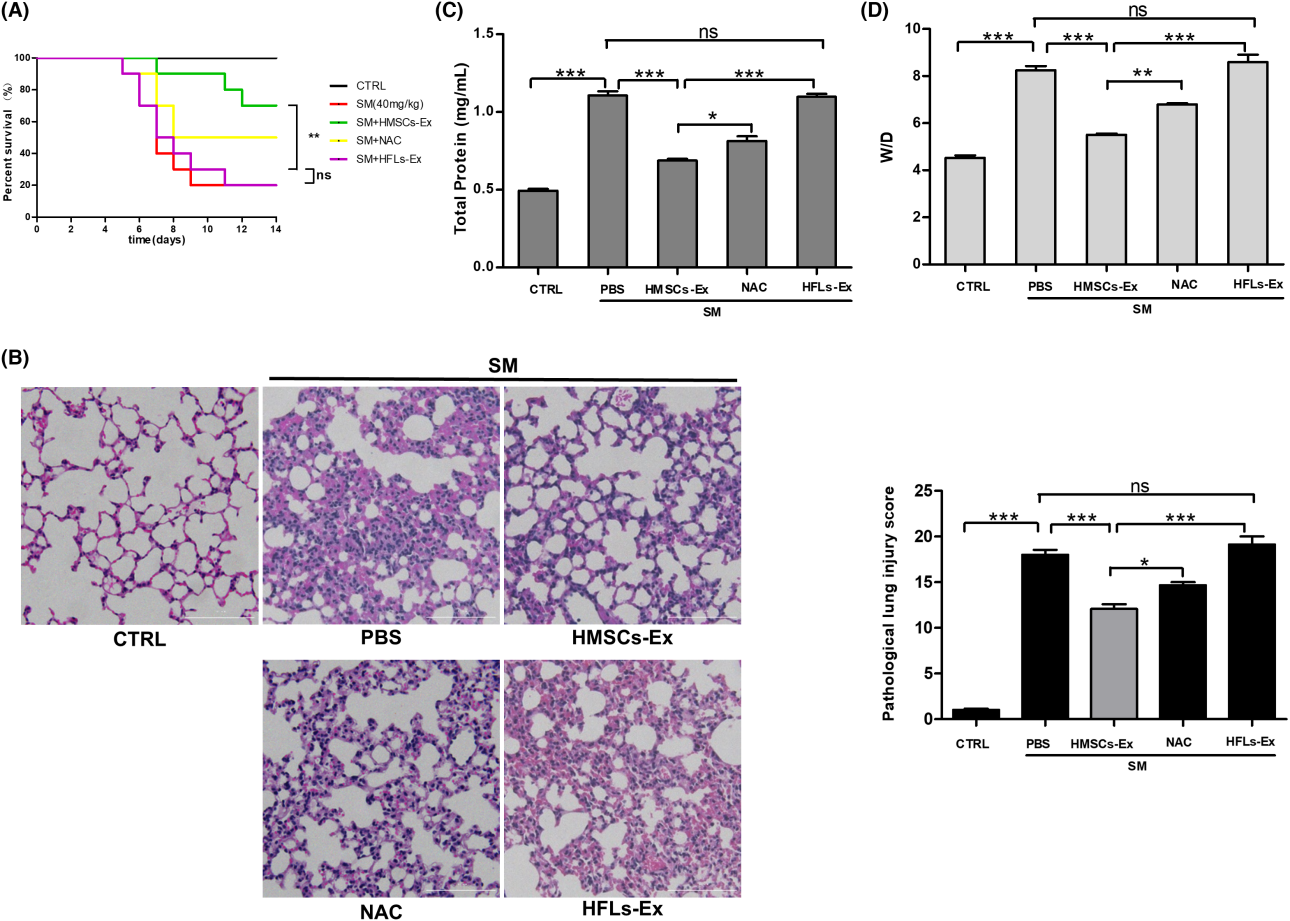
HMSCs‐Ex rescued SM‐induced lung injury in vivo. (A) Survival curves of SM‐injured mice treated with HMSCs‐Ex (3 × 10^8^ particles, resuspended in 150 μL of PBS) by tail vein injection. HMSCs‐Ex administration protected recipient animals from lung injury (*n* = 8; **p < 0.01). (B) Representative images of H&E‐stained lung tissues from SM‐injured mice after PBS, HFLs‐Ex or HMSCs‐Ex treatment. Original magnification: ×400. The HMSCs‐Ex group revealed conspicuous protection against SM‐injured lung damage, as showed by the relatively normal alveolar cavity, mucosal epithelium, and airways as well as minimal inflammatory cell infiltration and septal thickening. Comparison of pathological lung injury scores in SM‐exposed mice (*n* = 3; *p < 0.05; **p < 0.01; ***p < 0.001 compared with the SM group). (C and D) BALF protein levels (C) and wet‐to‐dry lung weight ratios (D) are reduced in HMSCs‐Ex administration group (*n* = 3; *p < 0.05; **p < 0.01; and ***p < 0.001). CTRL, control; HFLs‐Ex, human lung fibroblasts‐derived; HMSCs‐Ex, HMSCs‐derived exosomes; NAC, *N*‐acetylcysteine, PBS, phosphate‐buffered saline; SM, sulfur mustard.

An in vivo stable and optimized SM‐exposed mouse model was established as described previously to assess the therapeutic potential of HMSCs‐Ex.^17^ HMSCs‐Ex were isolated and qualified, as shown in Figure S1. Human lung fibroblasts‐derived exosomes (HFLs‐Ex) were used as controls. The five experimental groups were treated with propanediol solution, SM + phosphate‐buffered saline (PBS), SM + HMSCs‐Ex, SM + *N*‐acetylcysteine (NAC) and SM + HFLs‐Ex. SM treatment induced a decrease in dietary intake and physical activity after 24 h. On the third day, severe weight and appetite reduction occurred in the SM‐treated mice. The mortality rate of mice gradually increased until the fifth day. The body weight of the mice began to increase after Day 5, and no mice died since Day 8. Compared with the control (CTRL) group, the survival rate of the SM‐exposed mice decreased significantly, while HMSCs‐Ex could reverse the decrease in the survival rate (Figure 1A). These data revealed that the survival rate of SM‐exposed mice improved after treatment with HMSCs‐Ex; also, it could improve the general health status of the SM‐exposed mice.

Haematoxylin and eosin (H&E) staining was applied to investigate the pathological lesions in lung sections. After exposure to SM, the mice in the HMSCs‐Ex group had fewer tissue lesions and perivascular infiltrates compared with mice in the HFLs‐Ex and SM groups, indicating an impressive protective effect of HMSCs‐Ex. In addition, less bleeding and oedema were also observed in mice treated with HMSCs‐Ex (Figure 1B). The histological score was higher in the SM group than in the CTRL group (*p* < 0.05) but predominantly decreased in the HMSCs‐Ex group. In addition, the protein levels in the bronchoalveolar lavage fluid (BALF) and the wet‐to‐dry weight ratio (W/D ratio) of the lung tissues increased significantly in the SM group compared to the CTRL group, indicating increased exudation of pulmonary oedema (Figure 1C,D). The total protein in BALF and the W/D ratio significantly reduced in the HMSCs‐Ex group compared with the NAC group, while the HFLs‐Ex administration showed no significant effect. The protein in BALF diminished significantly in HMSCs‐Ex administration compared with the SM group, suggesting that the alveolar capillary permeability was enhanced and at the same time, the infiltration of inflammatory cells was mitigated in the lung tissues. These results indicated that HMSCs‐Ex therapy could attenuate alveolar capillary permeability, reduce inflammatory cell migration and therefore play an effective role in ameliorating SM‐induced lung injuries.

### 3.2 HMSCs‐Ex ameliorates SM‐induced lung oxidative injury.

**FIGURE 2:**
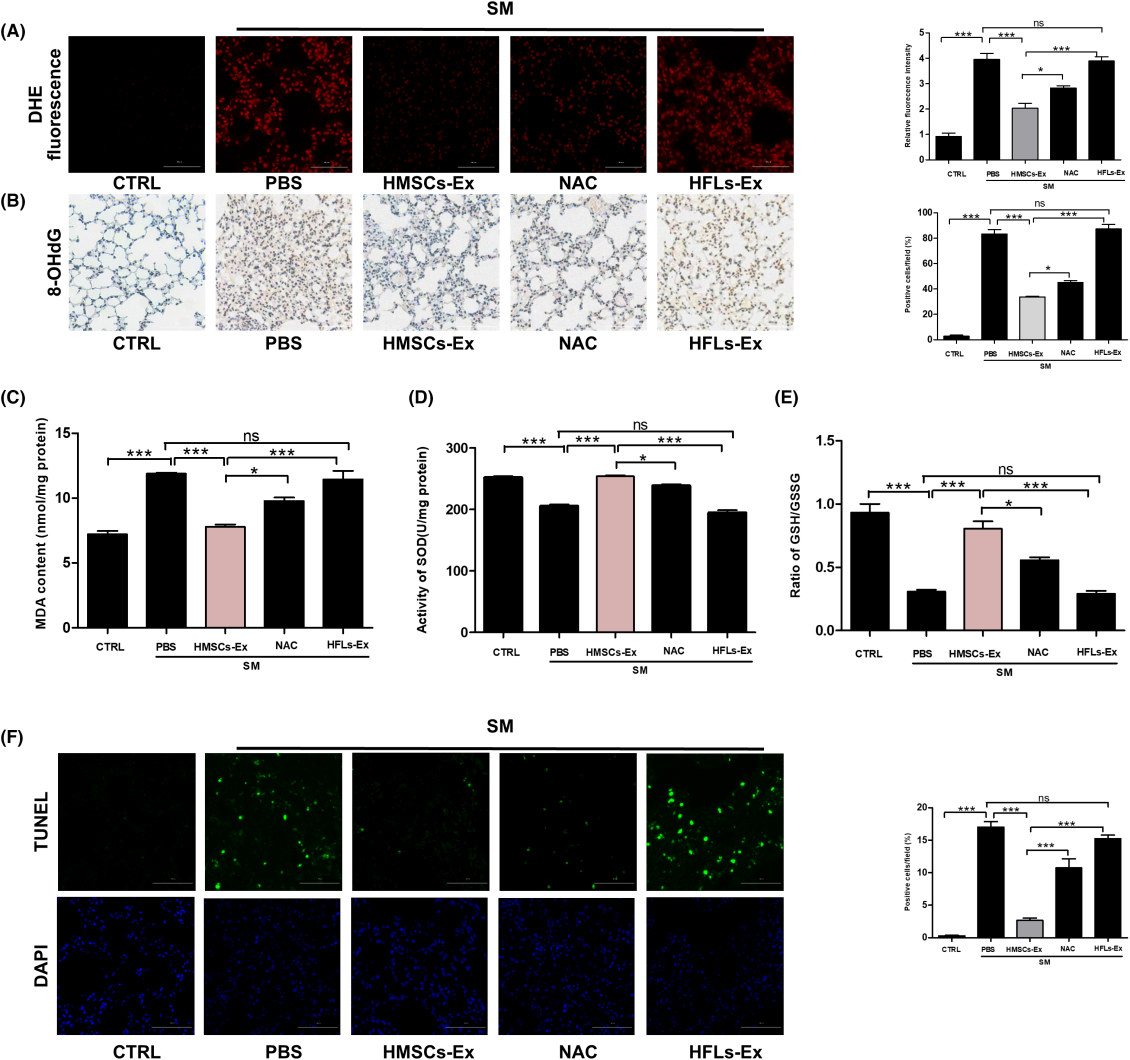
HMSCs‐Ex ameliorates SM‐induced lung oxidative injury. (A) DHE‐stained lung tissue slides from SM‐injured mice after HMSCs‐Ex or NAC treatment. Original magnification: ×400. Data showed the relative DHE fluorescence intensity in lung tissues from different groups. Values are the mean ± SEM (*n* = 3; *p < 0.05; and ***p < 0.001). (B) Immunohistochemical staining of mouse lung tissue slides; reduced oxidative DNA damage marker 8‐OHdG expression was found in HMSCs‐Ex group (*n* = 3; *p < 0.05; **p < 0.01; and ***p < 0.001). Original magnification: × 400. (C) Lipid peroxidation injury marker MDA content was detected as oxidative stress parameters. Data show that HMSCs‐Ex treatment markedly reduced MDA content (*n* = 3; *p < 0.05; **p < 0.01; and ***p < 0.001). (D and E) SOD activity (D) and GHS/GSSG ratio (E) in mouse lungs were measured. HMSCs‐Ex treatment significantly increased antioxidant enzyme content (*n* = 3; *p < 0.05; **p < 0.01; and ***p < 0.001). (F) TUNEL staining of mouse lung tissue slides; reduced apoptosis‐positive cells was found in HMSCs‐Ex group after treatment (*n* = 3; *p < 0.05; **p < 0.01; and ***p < 0.001). Original magnification: × 400. CTRL, control; HFLs‐Ex, human lung fibroblasts‐derived; HMSCs‐Ex, HMSCs‐derived exosomes; NAC, *N*‐acetylcysteine, PBS, phosphate‐buffered saline; SOD, superoxide dismutase; SM, sulfur mustard.

The increased production of reactive oxygen species (ROS) that induce oxidative stress and cell death through lipid peroxidation, protein oxidation, and DNA damage is one of the main mechanisms involved in SM toxicity. We investigated the effect of HMSCs‐Ex on SM‐induced intracellular ROS production using DHE staining. Fluorescence microscopy revealed an increase in ROS levels in the SM and HFLs‐Ex groups and a markedly decrease in the HMSCs‐Ex group (Figure 2A), indicating lower superoxide production. The oxidative DNA injury and lipid peroxidation injury in the lungs were determined by detecting the level of 8‐hydroxydeoxyguanosine (8‐OHdG) and malondialdehyde (MDA), respectively. As shown in Figure 2B,C, the 8‐OHdG content and MDA activity were higher in the SM group compared to the CTRL group, while the administration of HMSCs‐Ex effectively reversed the upregulation trend. The treatment with HMSCs‐Ex improved the activity of superoxide dismutase (SOD) and glutathione (GSH) level (Figure 2D,E). Terminal deoxynucleotidyl transferase enzymatic dUTP nick end labelling (TUNEL) staining, which labels 3′‐OH ends of DNA with ribonuclease, was conducted to assess extensive DNA degradation in apoptotic cells during late stages of apoptosis. The number of TUNEL‐positive cells in the lungs increased in the SM group, while HMSCs‐Ex treatment blocked the changes significantly (Figure 2F). Overall, these findings indicated that the treatment with HMSCs‐Ex could alleviate SM‐induced oxidative injury, promote antioxidant capacity, inhibit apoptosis in SM‐exposed mice and promote lung recovery.

### 3.3 HMSCs‐Ex suppressed SM‐induced oxidative stress via regulating the NRF2 signalling pathway in pneumonocyte

Using cell model of human normal lung epithelium cell line exposed to SM as previously reported,^22^, ^23^ we explored the function of HMSCs‐Ex in vitro. BEAS‐2B cell model exposure concentration and the effect of HMSCs‐Ex on pneumocyte survival was detected using Cell Counting Kit‐8 (CCK‐8) method, as shown in Figures S2 and 3A. Significant increases in the cell viability of SM‐exposed BEAS‐2B cells were observed in the HMSCs‐Ex groups compared with the HFLs‐Ex group (*p* < 0.01). The result revealed that the group treated with concentration of 1 × 10^9^ particles resuspended in 150 μL of PBS produced the most optimal effect.

**FIGURE 3:**
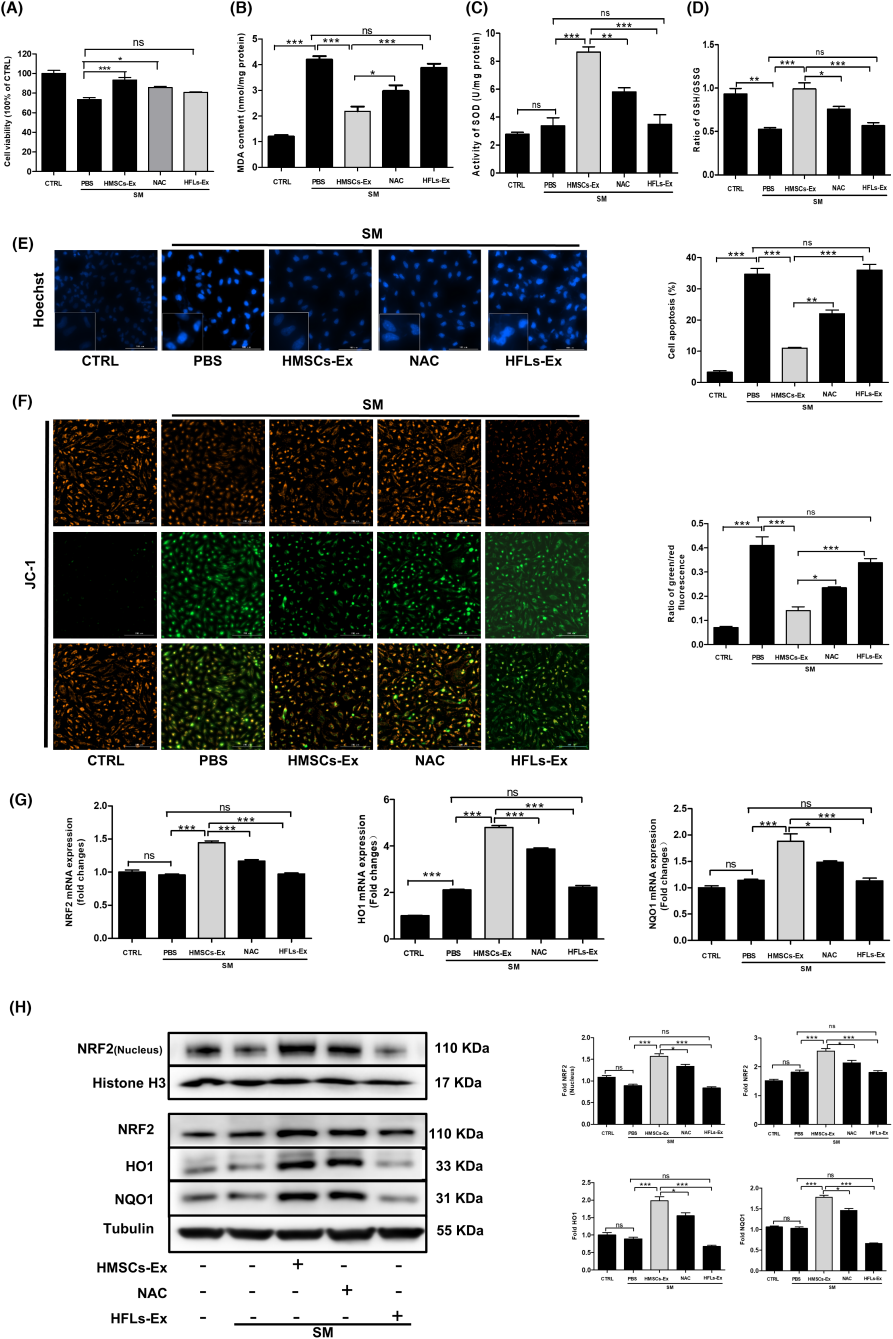
HMSCs‐Ex suppressed SM‐induced oxidative stress via regulating the NRF2 signalling pathway in pneumonocyte. (A) The effect of HMSCs‐Ex on pneumocytes survival was assessed by CCK‐8 assays. Data indicated that significant increases in the cell viability were observed in the HMSCs‐Ex treatment. (B–D) Intracellular MDA (B), SOD (C) and GSH/GSSG (D) content were measured, and HMSCs‐Ex treatment reduced the MDA release and increased the level of GSH and SOD activity (*n* = 3; *p < 0.05; **p < 0.01; and ***p < 0.001). (E) Hoechst 33342 staining of apoptotic SM‐injured BEAS‐2B cells cultured with PBS, NAC, HFLs‐Ex or HMSCs‐Ex for 24 h. HMSCs‐Ex treatment reduced BEAS‐2B cell apoptosis compared with PBS and HFLs‐Ex groups (*n* = 3; *p < 0.05; **p < 0.01). Original magnification: ×200. (F) Mitochondrial membrane potentials in PBS, HFLs‐Ex, and HMSCs‐Ex groups. Increases in green fluorescence indicate perturbed membrane potentials (*n* = 3; **p < 0.01). HMSCs‐Ex blocked the increase ratio of green to red fluorescence by SM treatment. Original magnification: ×400. (G) Expression of oxidative stress‐related mRNA in SM‐exposed BEAS‐2B cells was measured with qRT‐PCR 24 h after PBS, NAC, HFLs‐Ex or HMSCs‐Ex treatment. NRF2, HO1 and NQO1 mRNA levels were increased in the HMSCs‐Ex group. (H) Western blot analysis of oxidative stress‐related proteins in SM‐exposed BEAS‐2B cells treated with PBS, NAC, HFLs‐Ex or HMSCs‐Ex for 24 h. The levels of total NRF2, HO1, NQO1 and nuclear NRF2 were quantitated by densitometric analysis using ImageJ software and normalized to Tubulin shown as numbers under individual blots (*n* = 3; *p < 0.05; **p < 0.01; and ***p < 0.001). CTRL, control; HFLs‐Ex, human lung fibroblasts‐derived; HMSCs‐Ex, HMSCs‐derived exosomes; NAC, *N*‐acetylcysteine, PBS, phosphate‐buffered saline; SOD, superoxide dismutase; SM, sulfur mustard.

The levels of MDA, SOD and GSH in BEAS‐2B cells were detected to assess the oxidative stress damage and the anti‐oxidative enzyme production. BEAS‐2B cells were exposed to SM for 30 min and then treated with HMSCs‐Ex or NAC or HFLs‐Ex for 24 h. The MDA level (Figure 3B) significantly increased, while the SOD (Figure 3C) and GSH (Figure 3D) levels decreased drastically in the SM group. However, the administration of HMSCs‐Ex reduced the increase of MDA level and raised the levels of GSH and SOD.

Next, the anti‐apoptotic ability of HMSCs‐Ex was evaluated using Hoechst and JC‐1 staining. The results showed that fewer apoptotic cells and less chromatin condensation were detected in the HMSCs‐Ex‐treated group (Figure 3E) compared with those of the SM and HFLs‐Ex groups. The oxidative environment was associated with the early stages of apoptosis by lowering the mitochondrial membrane potential (ΔΨmito).^24^, ^25^ A special fluorescent cationic dye JC‐1 was used to detect the changes in mitochondrial membrane potential. It was evidenced that the mitochondrial depolarization was featured by the decrease in the red to green fluorescence intensity ratio. As shown in Figure 3F, the ratio of red to green fluorescence in the SM group strongly decreased, while HMSCs‐Ex blocked the changes significantly. These results revealed that HMSCs‐Ex possessing better efficacy in inhibiting oxidative stress–induced apoptosis in BEAS‐2B cells and protecting against SM‐induced injury compared with HFLs‐Ex and NAC.

In the pathogenesis of SM diseases, NRF2 is regarded as a critical transcriptional regulator of oxidative stress response.^23^ The mRNA levels of NRF2 and downstream regulators Haem‐Oxygenase‐1 (HO1) and NADPH quinone oxidoreductase 1 (NQO1) were examined in SM‐exposed BEAS‐2B cells from different groups using quantitative reverse transcription–polymerase chain reaction (qRT‐PCR) to confirm the role of HMSCs‐Ex in inhibiting oxidative stress. The results (Figure 3G) indicated that the downregulation of NRF2, HO1 and NQO1 mRNA levels was confirmed in the SM group, which was reversed by the HMSCs‐Ex administration. Further, we performed western blot to detect the changes in oxidative stress‐related proteins. It was revealed that NRF2 was translocated from the cytoplasm to the nucleus after the administration of HMSCs‐Ex, and the HO1 and NQO1 protein expression levels decreased after SM injury compared with the CTRL group; the administration of HMSCs‐Ex increased the expression (Figure 3H). Compared with HMSCs‐Ex, no effect was observed on the levels of oxidative stress‐related proteins in the treatment of HFLs‐Ex after SM stimulation. In summary, HMSCs‐Ex suppressed SM‐induced oxidative stress and apoptosis, which might contribute to the regulation of NRF2 protein translation.

### 3.4 HMSCs‐Ex suppressed the oxidative stress by transferring MiR‐199a‐5p

**FIGURE 4:**
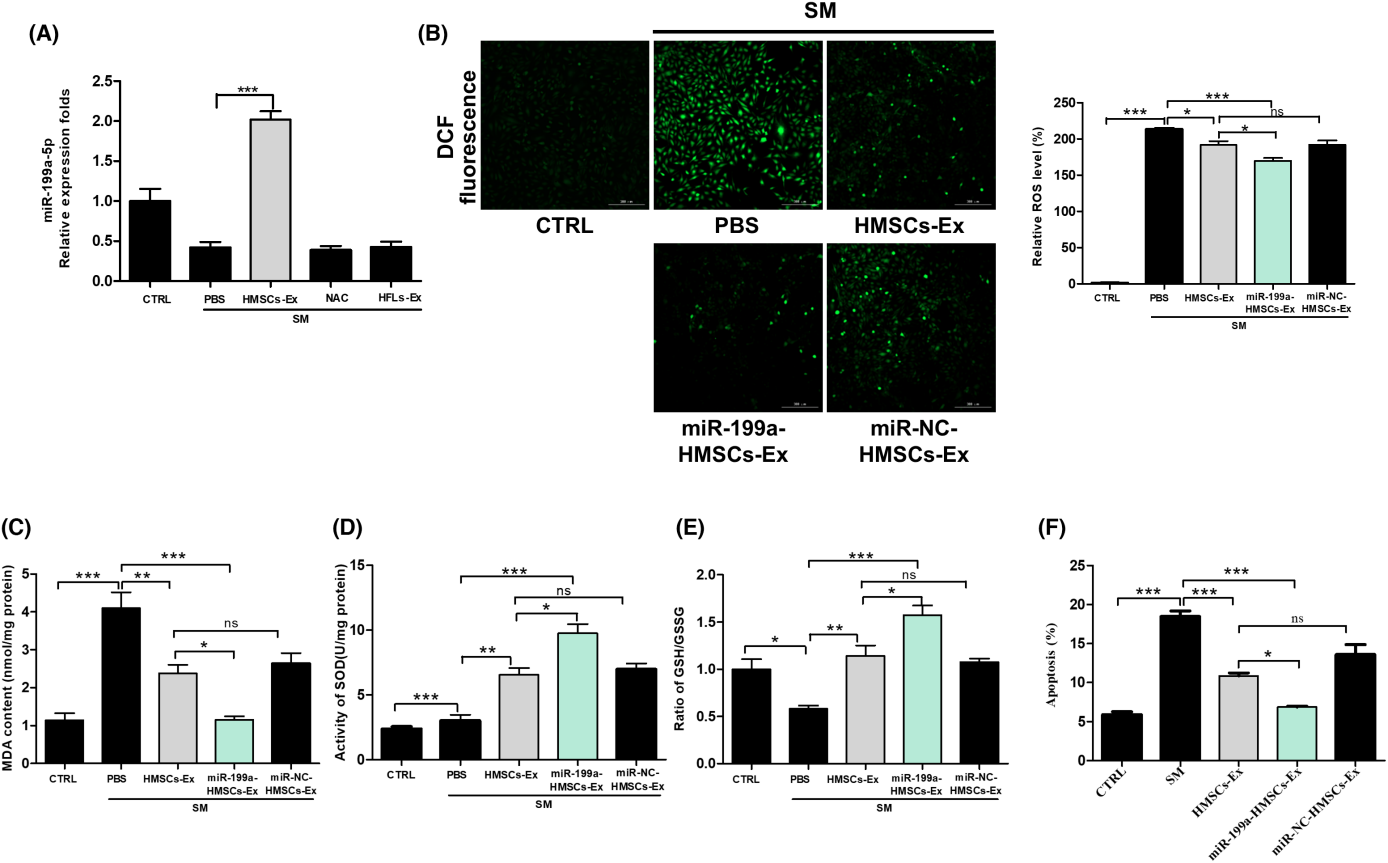
HMSCs‐Ex suppressed the oxidative stress by transferring MiR‐199a‐5p. (A) Quantitative analysis of relative miR‐199a‐5p levels in BEAS‐2B cells after the indicated treatment for 24 h (*n* = 3; ***p < 0.001). (B) ROS production in SM‐injured BEAS‐2B cells detected by DCF probe staining after treatment with PBS, HMSCs‐Ex, miR‐NC‐HMSCs‐Ex or miR‐199a‐HMSCs‐Ex. Representative images of DCF fluorescence in SM‐injured BEAS‐2B cells and relative DCF fluorescent values showed reduced ROS production in miR‐199a‐HMSCs‐Ex treated BEAS‐2B cells (*n* = 3; *p < 0.05; ***p < 0.001). (C–E) Intracellular MDA, SOD and GSH content were measured, and miR‐199a‐HMSCs‐Ex group treatment reduced the MDA release and increased the level of GSH and SOD activity compared with HMSCs‐Ex and miR‐NC‐ HMSCs‐Ex groups (*n* = 3; *p < 0.05; **p < 0.01; and ***p < 0.001). (F) The induction of apoptosis in BEAS‐2B cells was determined by Annexin V/PI double staining and flow cytometry. MiR‐199a‐Ex incubation decreased SM‐induced BEAS‐2B cell apoptosis compared with HMSCs‐Ex and miR‐NC‐HMSCs‐Ex groups (*n* = 3; *p < 0.05; **p < 0.01; and ***p < 0.001). The quantification of apoptotic cells is presented as the percent of total cells. CTRL, control; GSH, glutathione; HFLs‐Ex, human lung fibroblasts‐derived; HMSCs‐Ex, HMSCs‐derived exosomes; MDA, malondialdehyde; NAC, *N*‐acetylcysteine, PBS, phosphate‐buffered saline; ROS, reactive oxygen species; SOD, superoxide dismutase; SM, sulfur mustard.

Exosomes are membrane vesicles that can deliver specific functional components among which miRNA is the main form of functional RNA in exosomes.^16^, ^26^ To further understand the potential active component of HMSCs‐Ex in mediating the antioxidant effects, we analysed the inhibitors of ten of the most abundant miRNAs according to the exosomal miRNA sequencing results and the effect of the miRNAs on pneumocyte survival after SM exposure.^12^, ^27^, ^28^ Among these inhibitors, significant decreases were found in the cell viability rates in the miR‐199a‐5p inhibitor group compared with the SM group (Figure S3). Next, we detected the relative miR‐199a‐5p expression in SM‐exposed BEAS‐2B cells using qRT‐PCR and found that miR‐199a‐5p was selectively upregulated in the HMSCs‐Ex group and downregulated in the SM group (Figure 4A). These data suggested that miR‐199a‐5p might be the main functional component in regulating SM‐induced oxidative stress and apoptosis. We established miR‐199a‐HMSCs‐Ex overexpressed model by transfecting HMSCs‐Ex with miR‐199a‐5p or negative control (miR‐NC) to verify the function of miR‐199a‐5p in alleviating oxidative responsiveness in HMSCs‐Ex (Figure S4).

We then compared the effect of miR‐199a‐HMSCs‐Ex in alleviating oxidative responsiveness with that of miR‐NC‐HMSCs‐Ex. ROS levels were measured using a 2′,7′‐dichlorodihydrofluorescein diacetate (DCFH‐DA) probe. The fluorescent intensity showed that DCF‐positive staining (an indicator of ROS generation) was enhanced in the SM group compared with the CTRL group; the administration of miR‐199a‐HMSCs‐Ex induced an additional decrease in the percentage of DCF‐positive cells (Figure 4B). As shown in Figure 4C, the MDA level was lower in the miR‐199a‐HMSCs‐Ex group than in the miR‐NC‐HMSCs‐Ex group. The administration of miR‐199a‐HMSCs‐Ex exhibited better efficacy in increasing the activity of SOD and GSH than that of miR‐NC‐HMSCs‐Ex (Figure 4D,E). The anti‐apoptotic effects of miR‐199a‐HMSCs‐Ex in SM‐exposed BEAS‐2B cells were measured by Annexin V/propidium iodide (FITC‐AV/PI) double‐staining. We found that after 24 h treatment, the number of apoptotic cells diminished in the miR‐199a‐HMSCs‐Ex group compared with the SM and HMSCs‐Ex groups (Figures 4F and S5). The aforementioned results indicated that miR‐199a‐5p played a critical role in alleviating oxidative responsiveness and inhibiting apoptosis in HMSCs‐Ex.

### 3.5 HMSCs‐Ex‐derived miR‐199a‐5p activated the NRF2 signalling pathway by targeting CAV1

**FIGURE 5:**
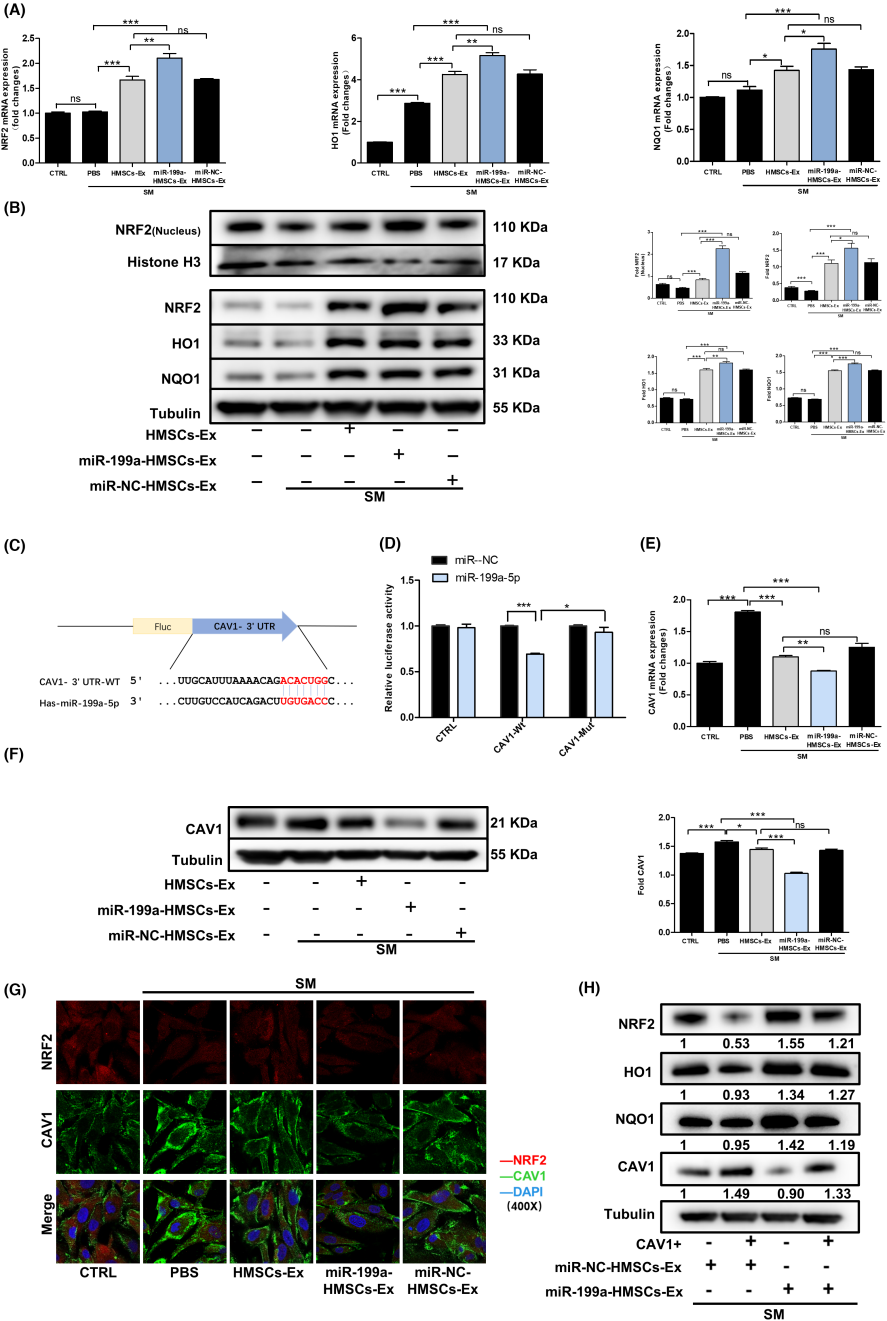
HMSCs‐Ex‐derived miR‐199a‐5p activated the NRF2 signalling pathway by targeting CAV1. (A) Expression of oxidative stress‐related mRNA in SM‐exposed BEAS‐2B cells was measured with qRT‐PCR 24 h after PBS, HMSCs‐Ex, miR‐NC‐HMSCs‐Ex or miR‐199a‐HMSCs‐Ex treatment. NRF2, HO1 and NQO1 mRNA levels were increased in the miR‐199a‐HMSCs‐Ex group. The miR‐199a‐HMSCs‐Ex treatment led to a marked increasement in HO1, NQO1 and NRF2 mRNA levels compared with miR‐NC‐HMSCs‐Ex. (*n* = 3; *p < 0.05; **p < 0.01; and ***p < 0.001). (B) Western blot analysis showed total NRF2, HO1, NQO1 and nuclear NRF2 protein expression changed accordingly with the corresponding mRNA levels. Quantitative analysis for relative total NRF2, HO1, NQO1 and nuclear NRF2 levels after the indicated treatment for 24 h (*n* = 3; *p < 0.05; **p < 0.01; and ***p < 0.001). (C) The binding site between miR‐199a‐5p and CAV1 3′UTR was analysed by TargetScan website. (D) The relationship between miR‐199a‐5p and CAV1 was validated by dual‐luciferase reporter assay. (E) Quantitative analysis of relative CAV1 mRNA expression in BEAS‐2B cells with qRT‐PCR 24 h after treated with PBS, HMSCs‐Ex, miR‐NC‐HMSCs‐Ex or miR‐199a‐HMSCs‐Ex (*n* = 3; *p < 0.05; **p < 0.01; and ***p < 0.001). CAV1 expression decreased in miR‐199a‐HMSCs‐Ex treated BEAS‐2B cells compared with HMSCs‐Ex and miR‐NC‐HMSCs‐Ex groups. (F) Western blot quantification of CAV1 expression in SM, HMSCs‐Ex, miR‐NC‐HMSCs‐Ex or miR‐199a‐HMSCs‐Ex. CAV1 protein was highly expressed in SM groups compared with HMSCs‐Ex, miR‐NC‐HMSCs‐Ex and miR‐199a‐HMSCs‐Ex groups. (G) Confocal laser scanning immunofluorescence analysis to detect the interaction between CAV1 and NRF2 expression in BEAS‐2B cells after the indicated treatment for 24 h. Original magnification: ×400 (*n* = 3; *p < 0.05; **p < 0.01; and ***p < 0.001). (H) Western blot quantification of CAV1, NRF2, HO1 and NQO1 expression in wild‐BEAS‐2B cells and CAV1‐overexpressed BEAS‐2B cells. CAV1 overexpression weakened the miR‐199a‐HMSCs‐Ex mediated promotion of the nuclear translocation of NRF2 and the expression of HO1 and NQO1. Data are expressed as relative ratios of specific proteins to Tubulin shown as numbers under individual blots (*n* = 3; *p < 0.05). CTRL, control; HFLs‐Ex, human lung fibroblasts‐derived; HMSCs‐Ex, HMSCs‐derived exosomes; NAC, *N*‐acetylcysteine, PBS, phosphate‐buffered saline; SM, sulfur mustard.

To explore the mechanism by which HMSC‐Ex‐derived miR‐199a‐5p suppressed oxidative stress, we examined both mRNA and protein expression levels of NRF2, HO1 and NQO1 in SM‐exposed BEAS‐2B cells from different groups using qRT‐PCR and western blot. The mRNA (Figure 5A) and protein (Figure 5B) levels of NRF2, HO1 and NQO1 remarkably decreased in the SM‐exposed BEAS‐2B cells compared with the CTRL cells. The administration of miR‐199a‐HMSCs‐Ex significantly upregulated NRF2, HO1 and NQO1 protein expression after 24 h. A consistent result was obtained in the relative quantification of protein expression of NRF2, HO1 and NQO1. The above results indicated that HMSC‐Ex‐derived miR‐199a‐5p played a significant role in NRF2 signalling pathway activation.

MiRNAs, downregulating gene expression at the transcriptional or posttranscriptional level, play important roles in a variety of biological processes.^29^ We investigated the potential targets of miR‐199a‐5p in order to explore its functional mechanism. As shown in Figure 5C, the bioinformatics website (TargetScan) indicated that miR‐199a‐5p could bind to the CAV1 mRNA 3′‐untranslational regions (3′‐UTR) by sequence alignment.

The miR‐199a‐5p and CAV1 targeting relationship was verified by dual‐luciferase reporter assay, as shown in Figure 5D. Previous reports also demonstrated that 3′‐UTR of CAV1 could be bound by miR‐199a‐5p and its protein level could be downregulated as well.^30^, ^31^ The effect between CAV1 and miR‐199a‐5p was confirmed by detecting the CAV1 mRNA level using RT‐qPCR (Figure 5E) and protein level using western blot (Figure 5F), indicating that CAV1 might participate in lung oxidative damage repair mediated by miR‐199a‐HMSCs‐Ex. The immunofluorescence assay showed a similar result and verified that the CAV1 expression significantly increased after SM exposure. Besides, the administration of miR‐199a‐HMSCs‐Ex was found to dramatically reduce CAV1 expression and promote NRF2 activity by confocal microscopy (Figure 5G). These results indicated that the overexpression of miR‐199a‐5p in HMSCs‐Ex induced a further decrease of CAV1 and the activation of the NRF2 signalling pathway than HMSCs‐Ex treatment.

CAV1 inhibited the expression of antioxidant enzymes by direct interaction with NRF2 and subsequent inhibition of its transcriptional activity in BEAS‐2B cells.^32^ Our previous study showed that CAV1 was an oxidation‐related protein.^33^ Next, we sought to evaluate the role of CAV1 in recruiting NRF2 for modulating HO1 and NQO1 expression. Stable CAV1‐overexpressing cell lines were established and confirmed (Figure S6). As is shown in Figure 5H, CAV1 overexpression reversed the upregulatory effect of miR‐199a‐HMSCs‐Ex on NRF2 expression and its downstream target proteins HO1 and NQO1 in SM‐exposed BEAS‐2B cells. The total NRF2 protein level was upregulated in the miR‐199a‐HMSCs‐Ex administration group, and this increase could be reversed by CAV1 overexpression. Taken together, the above results suggested that CAV1 was an essential player in the negative regulation of the NRF2 pathway in miR‐199a‐HMSC‐Ex‐mediated lung protection.

### 3.6 HMSCs‐Ex‐derived miR‐199a‐5p attenuated SM‐induced oxidative stress by regulating CAV1/NRF2 signal pathway in vivo

**FIGURE 6:**
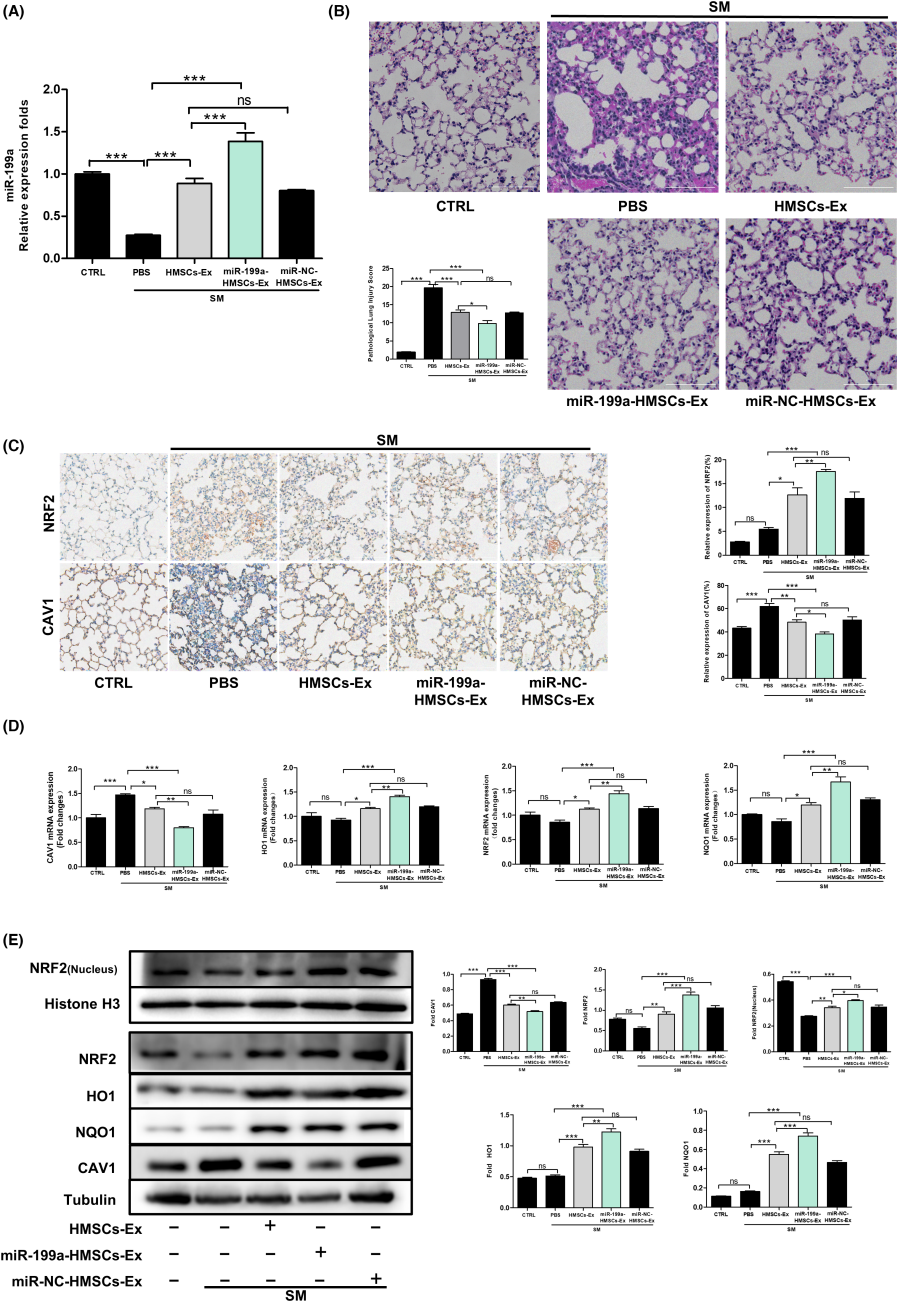
HMSCs‐Ex‐derived miR‐199a‐5p attenuated SM‐induced oxidative stress by regulating CAV1/NRF2 signal pathway in vivo. (A) Quantitative analysis of relative miR‐199a‐5p levels in lung sections of SM‐injured mice after treated with PBS, HMSCs‐Ex, miR‐NC‐HMSCs‐Ex or miR‐199a‐HMSCs‐Ex (*n* = 3; *p < 0.05; **p < 0.01; and ***p < 0.001). (B) Representative histological micrograph analysis of H&E staining of lung slides after the indicated treatment for 24 h. In addition, the quantitative assay is done using Image J software. Original magnification: ×400 (*n* = 3; *p < 0.05, **p < 0.01). (C) Immunohistochemical detection of positive NRF2 and CAV1 staining in the lung of SM‐expose mice 24 h after treated with PBS, HMSCs‐Ex, miR‐NC‐HMSCs‐Ex or miR‐199a‐HMSCs‐Ex (*n* = 3; *p < 0.05; **p < 0.01; and ***p < 0.001). (D) Quantitative analysis of relative CAV1, NRF2, HO1 and NQO1 in lung tissues was determined by qRT‐PCR (*n* = 3; *p < 0.05; **p < 0.01). (E) Protein levels of CAV1, total NRF2, HO1, NQO1 and nuclear NRF2 in lung tissues were determined by western blot analysis. Quantification of the protein expression in SM‐injured mice 24 h after indicated treatment (*n* = 3; *p < 0.05, **p < 0.01).

MiR‐199a‐HMSCs‐Ex or miR‐NC‐HMSCs‐Ex was intravenously injected into mice after SM exposure to evaluate whether HMSC‐Ex‐derived miR‐199a‐5p could serve as an approach to treat lung oxidative injury induced by SM in vivo. As expected, miR‐199a‐5p expression in lung decreased during SM treatment, and miR‐199a‐HMSCs‐Ex injection efficiently reversed the change in mice lungs (Figure 6A). H&E staining was used to analyse the morphologic alterations of the lung tissues. HMSCs‐Ex or miR‐NC‐HMSCs‐Ex treatment could improve the manifestations of pulmonary pathological damage such as diffuse interstitial oedema, alveolar air space reduction, alveolar thickening and leukocyte recruitment caused by SM exposure, while miR‐199a‐HMSCs‐Ex treatment further restored the recovery of lung tissue (Figure 6B). In addition, the histopathological damage score was used to assess the degree of lung damage.

CAV1 and NRF2 levels showed similar pattern as in vitro experiments (Figure 6C). The results from immunohistochemical assay indicated that the expression of CAV1 markedly reduced in the miR‐199a‐HMSCs‐Ex group compared with the HMSCs‐Ex or miR‐NC‐HMSCs‐Ex group, noticeably increased in the SM group in compare with the CTRL group. Meanwhile, NRF2 expression dramatically increased in the HMSCs‐Ex or miR‐NC‐HMSCs‐Ex group compared with the SM group, and markedly increased in the miR‐199a‐HMSCs‐Ex group compared with the HMSCs‐Ex or miR‐NC‐HMSCs‐Ex group. Consistent with our in vitro results, the RT‐PCR analysis showed that CAV1 mRNA levels significantly reduced in the miR‐199a‐HMSCs‐Ex group after 24 h while it obviously increased in the SM group. NRF2, HO1 and NQO1 mRNA levels significantly increased in miR‐199a‐HMSCs‐Ex group after 24 h compared with the HMSCs‐Ex group (Figure 6D). Western blot analysis showed the consistent result as is shown in Figure 6E. Taken together, the results accumulated in our experiments indicated that the administration of miR‐199a‐HMSCs‐Ex in the SM‐exposed mice significantly ameliorated lung oxidative injury by suppressing CAV1 expression and subsequently increasing NRF2 expression to promote the NRF2 pathway, implying the potential of miR‐199a‐5p as a treatment for lung injury in vivo.

## 4 DISCUSSION

Respiratory tract injury caused by SM is the leading cause of death from SM exposure. SM is a highly reactive bifunctional alkylating agent, rapidly reacting with a wide range of cellular components and molecular target spots. Oxidative stress, inflammatory response, alkylation of DNA and activation of proteolytic enzyme are all related to SM toxicity. NAC has a strong direct antioxidant activity and maintains the intracellular GSH level. Thus, it is recognized as the primary medicine candidate for treating pulmonary toxicity caused by SM.^34^ The injury caused by oxidative stress is the key link of acute and chronic SM damage.^20^ After SM exposure, oxidative stress damage occurs due to the mitochondrial deficiency, the increase of enzymes generated by ROS, and the consumption of glutathione and glutathione‐dependent antioxidant enzymes, causing the generation of ROS in the body to be unbalanced to the antioxidants content in the cells. Consequently, the DNA is damaged, which in turn leads to chromosomal abnormalities, altered gene expression, and genetic mutations, eventually resulting in cell death and tissue damage.^35^, ^36^ Therefore, seeking optimized medicine and methods for oxidative damage is the key to the treatment of mustard gas lung injury.

In the last few years, it has been proved that stem cell exosomes have beneficial effects on the treatment of acute respiratory distress syndrome (ARDS) and can exert stem cell–like effects. Previous studies found that BMSCs‐Ex had a therapeutic effect of boosting the repair of the epithelial barrier in pulmonary alveoli. However, neither the key components nor the mechanism of the actions was analysed. Exosomes mainly function by transducing components, including nucleic acids, proteins and so forth, in which miRNAs carried by the stem cell exosomes are the main components and have been confirmed to play a pivotal role in various damage repair functions of the mediating exosomes. MiRNAs modulate gene expression by suppressing the translation or stability of target gene sequences^37^ and are indispensible in various life processes such as cell differentiation, development and death.^38^, ^39^ MiRNAs sensitive to redox reactions have become a vital participant in the posttranscriptional regulation of redox‐mediated gene expression.^40^, ^41^

Increasing evidence suggests that certain miRNAs in the exosomes play a key role in the regulation of lung injury induced by oxidative stress and adjustment of the generation of ROS and endogenous antioxidant defence capabilities. For example, MSC‐derived exosomes alleviate ischemia/reperfusion injury in the lungs of mice through transporting anti‐oxidative stress and anti‐apoptotic miR‐21‐5p.^42^ They also inhibit the expression of P2X7 via transferred miR‐124‐3p, thereby suppressing the inflammatory response and ameliorating oxidative stress injury in traumatic ALI.^43^ In this study, we confirmed that HMSCs‐Ex had a significant inhibitory effect on SM‐induced acute lung oxidative damage and cell apoptosis, and identified exosome miR‐199a‐5p as the key component for its inhibitory effect on oxidative damage.

As an essential component of exosomes, miR‐199a‐5p participates in many cellular activities,^44^, ^45^ including cell proliferation,^46^ autophagy^47^ and regulation of angiogenesis.^48^ Interestingly, current studies on the functions of miR‐199a‐5p on lung physiology and disease showed controversial results. For example, Li et al. reported that the overexpression of miR‐199a‐5p inhibited the proliferation of non‐small cell lung cancer cells by arresting the cell cycle in the G1 phase.^49^ However, other data suggested that the downregulation of miR‐199a‐5p inhibited the expression of BclGs (Bcl‐Gonad short form) such as caspase‐3, Bax and Bcl‐2 in the alveolar macrophages as well as the upregulation of proinflammatory cytokines such as IL‐1, TNF‐α and IL‐6 in the mouse alveolar macrophage inflammation model and sepsis‐induced ARDS model.^50^ MiR‐199a‐5p promoted idiopathic pulmonary fibrosis and caused alternations in gene expression and cellular functions in pulmonary fibrosis.^31^ These findings suggested that, as a multifunctional miRNA, miR‐199a‐5p functioned differently in different cell types and stress situations.^51^ Some studies suggested that miR‐199a‐5p was subjected to the regulation by ROS. For example, Jun He et al. proposed that ROS generated by oxidative stress inhibited the expression of miR‐199a‐5p and further promoted the growth of ovarian tumour cells in humans.^52^ It was also reported that carcinogen arsenic in the environment induced the upregulation of ROS levels and the generation of oxidative stress, resulting in the loss of miR‐199a‐5p in human bronchial epithelial cells, which further promoted angiogenesis and tumour growth.^53^ In this study, we found that HMSCs‐Ex treatment promoted the increase of miR‐199a‐5p expression in BEAS‐2B cells. HMSCs‐Ex modified with overexpressed miR‐199a‐5p further promoted its efficacy in anti‐oxidative and anti‐apoptotic aspects. This indicated that miR‐199a‐5p is a key component of HMSCs‐Ex and participates in the anti‐oxidative action of HMSCs‐Ex. Further analysis found that the treatment with miR‐199a‐HMSCs‐Ex elevated the expression of NRF2 in the nucleus and the expression of downstream antioxidant enzymes HO1 and NQO1, indicating that the exosome miR‐199a‐5p mainly played its role in inhibiting oxidative damage by regulating the canonical NRF2 pathway.

How does miR‐199a‐5p achieve the regulation of NRF2 molecules? We further analysed the target molecules of miR‐199a‐5p acting in the cells. Bioinformatics analysis and literature proved that CAV1 might be the target molecule on which miR‐199a‐5p took effect.^30^, ^31^ Moreover, we used dual‐luciferase reporter gene analysis and verified that miR‐199a‐5p literally targeted CAV1 and downregulated its expression. We examined the relationship between CAV1 and exosomal miR‐199a‐5p in NRF2 signalling pathway. We found that CAV1 exhibited a larger decrease after the treatment with miR‐199a‐HMSC‐Ex than that of HMSCs‐Ex, and the miR‐199a‐HMSC‐Ex treatment showed a higher level of antioxidant enzymes expression, and had stronger resistance to oxidative stress‐induced cytotoxicity. However, the overexpression of CAV1 in BEAS‐2B cells attenuated the promoting effect of miR‐199a‐HMSCs‐Ex on SM‐induced activation of NRF2 signalling. In addition, the treatment with miR‐199a‐HMSCs‐Ex in vivo further reduced the expression of CAV1 in lung tissues.

CAV1 is the major resident scaffolding protein component of caveolae.^54^ It possesses a variety of biological functions in a wide range of cell types, including but not limited to regulation of vesicular trafficking, cholesterol homeostasis, proliferation and apoptosis.^55^ In pulmonary system, CAV1 functions as the major coat protein of the alveolus.^56^ CAV1 plays an important role in many human diseases. CAV1 knockout mice are more resistant to hyperoxia‐induced ALI,^57^, ^58^, ^59^ partly due to the enhanced antioxidant capacity. Increasing evidence indicates that CAV1 is of vital significance to NRF2 signalling. Previous report proved that in BEAS‐2B, CAV1 was a direct binding ally of NRF2 through the caveolin‐binding domain (amino 281–289) and could interplay with NRF2 directly, thereby affecting the binding of Keap1‐NRF2 and nuclear translocation of NRF2 (32). It has also been suggested that apart from the fact that the NRF2 signalling pathway is mediated by Keap‐1, CAV1 is also an endogenous inhibitor of NRF2 (33), and the interaction between CAV1 and NRF2 inhibits the transduction of the NRF2 signalling, resulting in the weakening of cellular antioxidant capacity. Consistent with these studies, we provided evidence that miR‐199a‐5p could directly inhibit CAV1 expression in lung epithelial cells, thus promoting the nuclear translocation of NRF2. This process induced the follow‐up expression of many downstream genes HO1 and NQO1 and the subsequent anti‐oxidative and anti‐apoptotic capacities of the cells. The anti‐apoptotic potential of HO1 has been fully understood.^60^ What's more, our in vivo experiment revealed that SM‐exposed mice treated with miR‐199a‐HMSC‐Ex had less lung injury, decreased CAV1 expression, increased NRF2 level and inhibited apoptosis.

In summary, our results showed that HMSCs‐Ex promoted the expression and nuclear import of NRF2 molecules through transducing miR‐199a‐5p and targeted binding with CAV1, thereby promoting the expression of antioxidant enzymes in lung cells and actively regulating SM‐induced oxidative stress.

## 5 CONCLUSIONS

In conclusion, we demonstrated the critical role of exosome‐mediated miR‐199a‐5p in regulating SM‐induced oxidative stress and apoptosis. We also expounded the precise mechanisms of how exosome‐mediated miR‐199a‐5p activated the CAV1/NRF2/HO1 axis and induced oxidative stress.

The study not only increased our understanding of exosome‐mediated treatment for SM but also contributed to the development of the strategy for the effective treatment of SM. However, the treatment often does not take effect due to exosomal miR‐199a‐5p alone. Besides miR‐199a‐5p, many other miRNA and protein components in exosomes should also contribute to the therapeutic effects of exosomes in lung injury caused by SM. With the development of exosomes transformed through an engineering approach, further efforts are required to study the biological parameters (optimal combination) and the treatment potential for multicomponent transduction to maximize the potential of medicine delivery.

## AUTHOR CONTRIBUTIONS

**Chuchu Gong:** Methodology (lead); writing – original draft (lead). **Zhengyan Gu:** Formal analysis (lead); investigation (equal). **Xinkang Zhang:** Investigation (lead); writing – review and editing (equal). **Qingqiang Xu:** Data curation (lead); funding acquisition (equal); writing – review and editing (lead). **Guanchao Mao:** Validation (lead). **Zhipeng Pei:** Formal analysis (supporting); funding acquisition (supporting). **Wenqi Meng:** Methodology (lead). **Jinfeng Cen:** Visualization (lead). **Jihao Liu:** Formal analysis (equal). **Xiaowen He:** Resources (equal). **Mingxue Sun:** Conceptualization (equal); funding acquisition (equal). **Kai Xiao:** Conceptualization (lead); funding acquisition (lead); resources (lead).

## FUNDING INFORMATION

This work was funded by the National Natural Science Foundation of China (82103885, 81871521, 81671858, 81803280), Natural Science Foundation of Shanghai (20ZR1470300, 21ZR1477700), the Shanghai Municipal Health Commission‐Outstanding Youth Foundation of Public Health (GWV‐10.2‐YQ48) and the Shanghai Sailing Program (21YF1457400).

## CONFLICT OF INTEREST STATEMENT

The authors declare that they have no conflict of interests or personal relationships that could have appeared to influence the work reported in this paper.

## CONSENT FOR PUBLICATION

All authors agree to be published. A preprint has previously been published (Chuchu Gong et al. 2022).

## Supporting information


Data S1.

## Data Availability

All data generated or analysed during this study are included in this published article.
