# Supplementary material for: HMSCs exosome‐derived miR‐199a‐5p attenuates sulfur mustard‐associated oxidative stress via the CAV1/NRF2 signalling pathway
Source: J Cell Mol Med. 2023 Jun 29;27(15):2165–82. doi: 10.1111/jcmm.17803 (PMC10399537; doi:10.1111/jcmm.17803)
Supplement: Supplementary file 1 — Data S1. [file JCMM-27-2165-s001.docx]

Additional file 1

**Characterization of HMSCs-Ex**

Exosomes are small phospholipid bilayer vesicles released by various types of cells [1]. HMSCs were identified based on morphological characteristics and differentiation capacity using flow cytometry assay and an inverted microscope. As shown in **Figure S1A**, the surface markers CD44, CD29, CD73 and CD166 were detected while the expression of CD34, CD45 and CD11b was negative. Red oil O staining, alizarin red staining, and toluidine blue staining were used to reveal the potentials of adipogenesis, osteogenesis, and chondrogenesis, respectively (**Figure S1B**)). These results are in accordance with the minimal criteria established by the International Society for Cellular Therapy in 2006 for characterizing multipotent mesenchymal stromal cells [2]. We isolated exosomes from the HMSCs supernatant to evaluate the function of HMSCs-Ex in lung injury induced by SM. Various methods to monitor the isolated exosomes were applied. First, the typical morphological features of phospholipid bilayer of the membrane were observed using transmission electron microscopy (**Figure S1C**). We next used a nanoparticle tracking analyzer (ZetaView, Particle Metrix, Inning am Ammersee, Germany) to detect the diameter distribution of the isolated exosomes samples accurately. **Figure S1D** showed the range of the diameter of the samples was from 40 to 160 nm and the single peak was around 100 nm. Western blot analysis was conducted to further verify the external and internal biomarkers of the exosome samples. To measure the internalization and distribution of exosomes in BEAS-2B cells, HMSCs-Ex were labeled with fluorescent carbocyanine dyes CM-Dil (red). After 4 h co-incubation, the uptake efficiency was measured by fluorescence microscopy, and the results showed that red fluorescence was detected in the BEAS-2B cell, suggesting that internalization of labeled exosomes had occurred (**Figure S1E**). **Figure S1F** showed that external (CD63) and internal biomarkers (HSP70 and TSG101) were abundant in the isolated samples. The above results suggest that the isolated HMSCs-Ex is qualified for subsequent experiments due to their high purity.

**
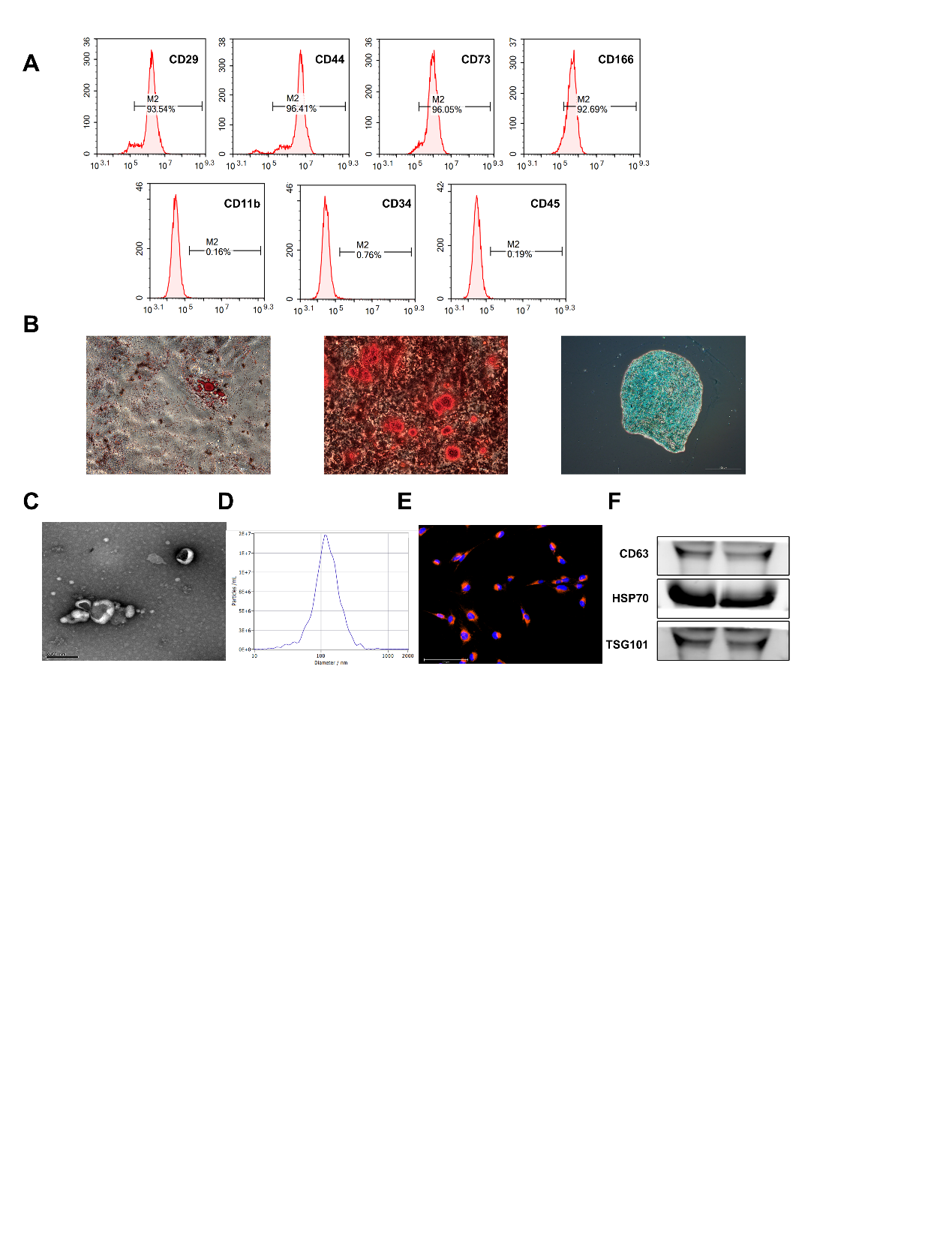
**

**Figure S1. Characterization of HMSCs-Ex. A** Immune phenotype of HMSCs positive for CD29, CD44, CD73, CD166 and negative for CD11b, CD34, CD45. **B** Oil red O staining of adipo-induced HMSCs, alizarin red staining of osteo-induced HMSCs and toluidine blue staining of chondro-induced HMSCs. **C** Transmission electron microscopy images of the main morphological characteristics of HMSCs-Ex, Scale bar=200 nm. **D** Nanoparticle tracking analyze of HMSCs-Ex distribution. The diameter of isolated HMSCs-Ex ranged from 40 to 160 nm, with a single peak at approximately 100 nm. **E** CM-Dil-labeled exosomes were taken up by BEAS-2B cells (scale bar = 75 μm). **F** Western blot quantification of markers of TSG101, HSP70, CD63 in HMSCs-Ex.

**Determination of** **BEAS-2B Cell Model Exposure Concentration.** BEAS-2B cells were exposed to increasing concentrations of SM respectively and the cell viabilities were then assayed using Cell Counting Kit-8 (CCK-8) method. The data showed that the cell viability of the SM-exposed cells was reduced in a dose-dependent manner (Figure **S2**). As the cell viability was decreased by approximately 20% in [culture](javascript:;) [medium](javascript:;) treated with 12.5μM SM for 24 h compared with that in CTRL [culture](javascript:;) [medium](javascript:;), the dose 12.5μM of SM was chosen in succeeding experiments.

**
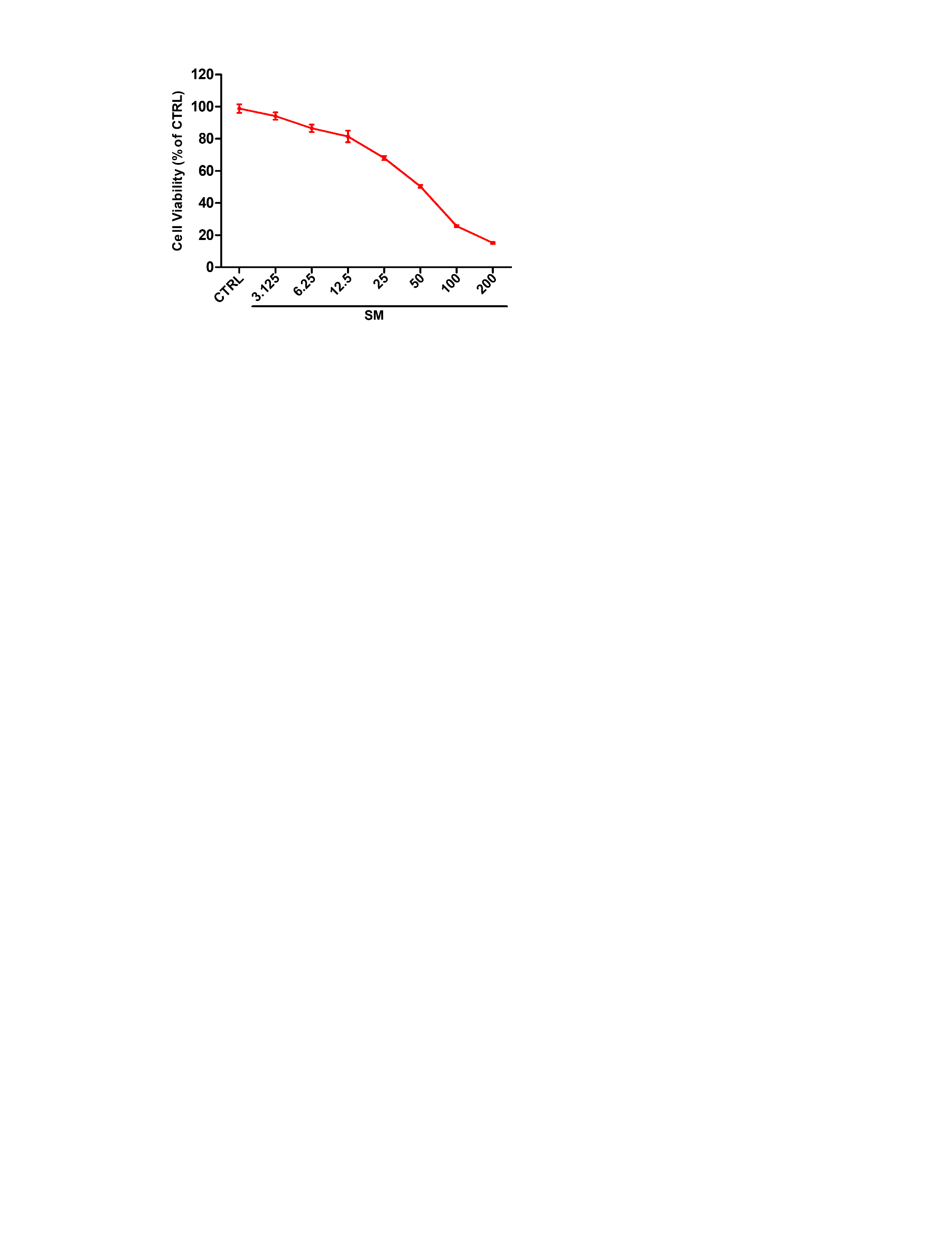
**

**Figure S2.** BEAS-2B cell viability was detected by CCK-8 assays.


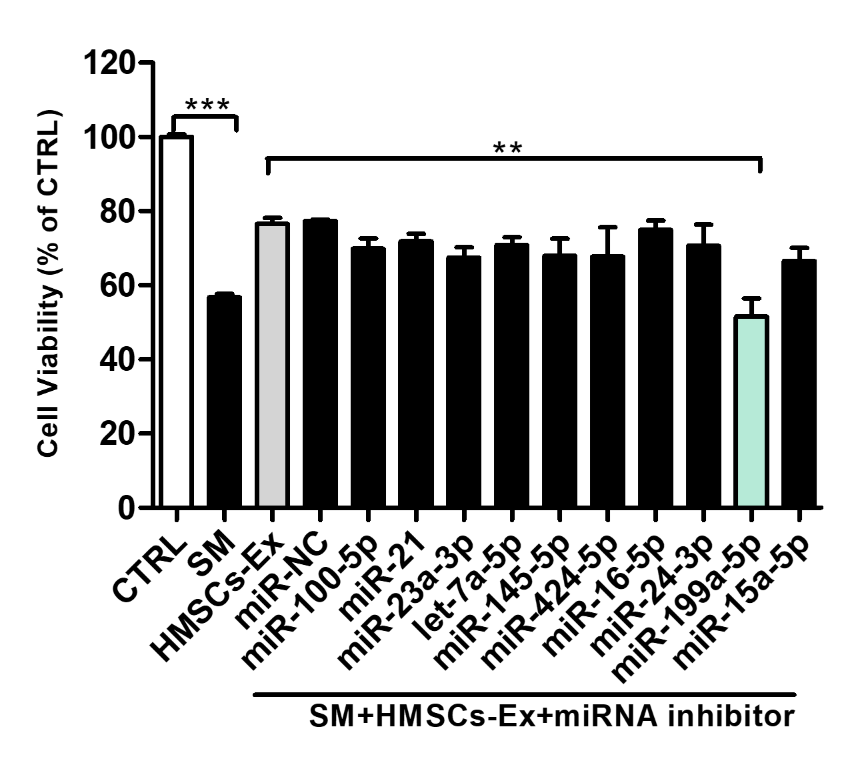


**Figure S3.** The efficiency of 10 potential active miRNA in HMSCs-Ex on pneumocytes survival was assessed by CCK-8 assays. Data indicated that miR-199a-5p might play a critical role in improving cell vitality. (n = 3; **P* < 0.05; ***P* < 0.01; and ****P* < 0.001.)

**Figure S4.** Quantitative analysis for relative miR-199a-5p expression in miR-NC- HMSCs-Ex and miR-199a-HMSCs-Ex group. The data confirmed that miR-199a-5p level increased upon miR-199a transfection (*n* = 3; **P* < 0.05; ***P* < 0.01; and ****P* < 0.001.).


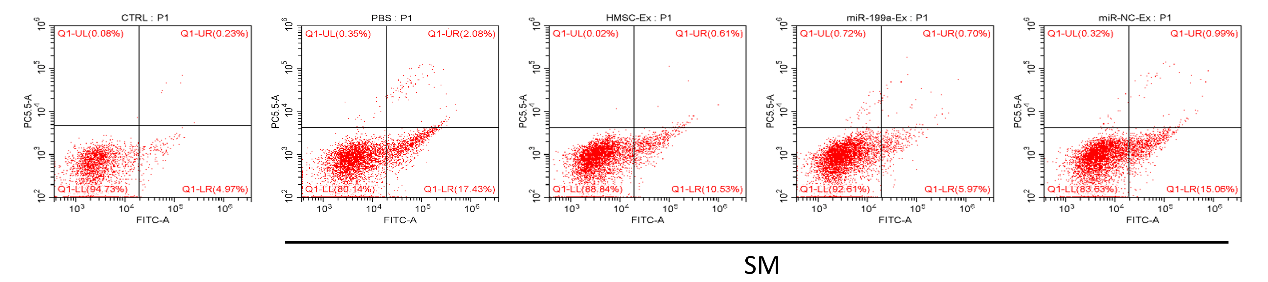


**Figure S5.** The induction of apoptosis in BEAS-2B cells was determined by Annexin V/PI double staining and flow cytometry. MiR-199a-HMSCs-Ex incubation decreased SM-induced BEAS-2B cell apoptosis compared with HMSCs-Ex and miR-NC-HMSCs-Ex groups. (*n* = 3; **P* < 0.05; ***P* < 0.01)


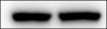

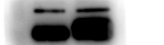


**Tubulin**

**CAV1**

**1 1.8**

**CAV1 Vector oe**

**Figure S6.** Detection of CAV1 protein expression in CAV1-overexpressed BEAS-2B cells by Western blot. oe is for CAV1 overexpression group.

**Supplementary references**

1. Kalluri R, LeBleu VS. The biology, function, and biomedical applications of exosomes. Science (New York, NY). 2020;367.

2. Dominici M, Le Blanc K, Mueller I, Slaper-Cortenbach I, Marini F, Krause D, et al. Minimal criteria for defining multipotent mesenchymal stromal cells. The International Society for Cellular Therapy position statement. Cytotherapy. 2006;8:315-7.
